# SARS-CoV-2 antibody progression and neutralizing potential in mild symptomatic COVID-19 patients – a comparative long term post-infection study

**DOI:** 10.3389/fimmu.2022.915338

**Published:** 2022-08-17

**Authors:** Jessica Brehm, Alexander Spaeth, Lars Dreßler, Thomas Masetto, Rainer Dannenberg, Christoph Peter, Matthias Grimmler

**Affiliations:** ^1^ MVZ Medizinische Labore Dessau Kassel GmbH, Dessau-Roßlau, Germany; ^2^ Institute of Molecular Medicine I, Medical Faculty, Heinrich Heine University Düsseldorf, Düsseldorf, Germany; ^3^ DiaSys Diagnostic Systems GmbH, Holzheim, Germany; ^4^ Hochschule Fresenius gGmbH, University of Applied Sciences, Idstein, Germany

**Keywords:** mild progression COVID-19, SARS-CoV-2, long-COVID, quantification immune response, long-term assay comparison, neutralizing potential

## Abstract

**Background:**

Since December 2019, SARS-CoV-2 has been keeping the world in suspense. Rapid tests, molecular diagnosis of acute infections, and vaccination campaigns with vaccines are building blocks of strategic pandemic control worldwide. For laboratory diagnostics, the quantification of the antibody titer of convalescents and vaccinated patients is thus increasingly coming to the fore.

**Methods:**

Here we present an evaluation on the comparability of five serological tests on a cohort of 13 patients with mild COVID-19 disease. Also participants who were vaccinated after recovery were included in this study. All common immune methods (ELISA, CLIA, PETIA) and SARS-CoV-2 specific antigens (N-, S1- and RBD-) were specifically tracked and directly compared for up to 455 days. The titer of recovered participants was also set to the degree of symptoms during infection and the occurrence of Long-COVID. In addition, relative comparability of different serological tests, all standardized to WHO, was set in reference to the neutralizing potential of the corresponding participants.

**Findings:**

The individual immune responses over 455 days after a mild SARS-CoV-2 infection remain stable, in contrast to vaccinated participants. All sero-tests reveal comparable performance and dynamics during the study and compared well to a surrogate neutralization test.

**Conclusion:**

The information presented here will help clinicians in the daily laboratory work in the selection and evaluation of different serological tests offered. The data also will support in respect of a sero-test-based neutralization cutoff.

## Introduction

In December 2019 the new *Severe Acute Respiratory Syndrome Coronavirus 2* (SARS-CoV-2) emerged in Wuhan, China, causing a devastating worldwide pandemic (1). SARS-CoV-2 infection can lead to the acute respiratory *Coronavirus Disease 2019* (COVID-19) which can display asymptomatic, mild, or severe progression (2). Up to now over 446 million confirmed COVID-19 cases and about 6 million deaths have occurred worldwide (data from John Hopkins University, March 20, 2022) (3).

While the acute infection is diagnosed by real-time reverse transcription-polymerase chain reaction (qRT-PCR) in respiratory samples, several assays have been developed to assess the serological status in individuals. Current serological tests quantify antibodies circulating in the blood of patients in response to the patient’s infection with the SARS-CoV-2 coronavirus (4–6). The dynamics of quantification of antibodies in regard to a SARS-CoV-2 infection can vary drastically upon patient-specific factors: the disease severity (asymptomatic – mild - severe), the rise and fall of associated immune globulin (Ig)-isotypes of a patient or his/her age, and respective immune status (7–10). The kinetics, the onset, and the progression of a SARS-CoV-2 immune response upon infection have not yet been conclusively investigated and compared for all methodical principles and antigens. In particular, the onset of antibodies and the seroconversion was described 10-14 days after the onset of symptoms (7). IgM and IgA class/isotypes of SARS-CoV-2 antibodies do appear earlier, followed by IgG. IgG class of antibodies can be detected much longer after the infection has subsided (11–13). In the case of SARS-CoV-2 comparatively early appearance of IgG antibodies was reported (14). Interestingly Moura et al. observed an increase of specific isotypes IgG1 and IgG3 already 8 days after onset of symptoms, while IgG4 levels overall were less detectable. Surprisingly, patients who died within 21 days after onset of symptoms also showed higher levels of IgG4, compared with recovered patients, suggesting that some life-threatened patients can elicit IgG4 to RBD antibody response in the first weeks of symptom onset. Specific IgG subtypes for this may be important as prognostic markers e.g., in predicting survival or sensitivity of patients to Long-COVID (15).

Quantification of antibodies also depends on the principle of the assay utilized including the used SARS-CoV-2 specific antigen. So far serological test principles of SARS-CoV-2 (ELISA, enzyme-linked immunosorbent assay; CLIA, chemiluminescence immunoassays, PETIA, particle-enhanced turbidimetric immunoassay) essentially differ by the detection of classes of antibodies. Assays do either individually detect specific isotypes of antibodies (IgG, IgM, IgA, IgE) or detect all classes of antibodies (5, 16). Spaeth et al. evaluated a variety of commercial assays and principles in regard of their kinetics, specificity and sensitivity upon patient-individual antibody serotype conversion (16). On the other hand, the viral protein selected to build the assay system is crucial to bind and detect a patient’s SARS-CoV-2 specific antibodies. An important aspect in this context is the degree of sequence concordance of the SARS-CoV-2 proteins with other viral proteins and the specificity of the available assays in regard of the seven known human pathogenic coronaviruses (HCoV). Four of these species circulate endemically worldwide (HCoV-229E, HCoV-NL63, HCoV-HKU1, and HCoV-OC43), predominantly causing mild colds but can also cause severe pneumonia in early childhood and elderly individuals (17–20). Available serological tests primarily utilize the viral nucleocapsid proteins (N), the spike protein (S), and the receptor-binding domain of the spike protein (RBD) of SARS-CoV-2 (5, 6, 21–23). The N-protein is the most abundant protein in SARS-CoV-2 (20). Antibodies to the viral N-protein decline faster than those to the receptor-binding domain or the entire spike protein, and therefore may substantially underestimate the proportion of SARS-CoV-2 exposed individuals (24). Besides clear limitations in the uses of N-based serological tests, some very recent reports describe its utilization in diagnostic settings and monitoring of SARS-CoV-2 (25, 26).

As neutralizing antibodies especially target the site of the RBD of the highly dynamic S protein, they are predesignated to induce protective immunity against viral infections (24, 27). The time point(s) of sampling and the selected kind of test for all of this has a crucial impact on quantification and the sensitivity and specificity of a test. It has been reported both that antibody titers vary with disease severity and that no differences in titer levels could be observed between severe and non-severe COVID-19 cases (7–10, 28–33). Furthermore, it has been shown that antibody titers decline rapidly, especially in mild and asymptomatic patients, while other studies report on stable antibody levels over several months (7, 9, 10, 34–38). High levels of neutralizing antibodies are good predictors for immune protection (39). However, reports differ regarding differences or changes in titer levels in mild vs. severe cases (40–44). The dynamics of SARS-CoV-2 infections are of particular interest in the management of the pandemics since the majority of the affected patient is mildly affected. This also will be of more importance due to the progression of the pandemic, especially when specific variants of concern (VOC) like the so-called omicron variants (line B.1.1.529, subtype BA.1 and BA.2), characterized by a higher rate of infection but less aggressive progression will further spread (45, 46). Several reports on the aspect of suitability of current sero-tests or neutralization assays in detecting antibodies generated by VOC strains are availably so far, indicating a diverse picture in the efficiency of assays to detect or neutralize variants of SARS-CoV-2 (44, 47–52). Overall, mildly affected patients so far are remarkably underrepresented in studies covering the diverse effects of the pandemic.

After a COVID-19 disease, it often takes several months for convalescents to get fit again. Even in those affected with a rather mild course of the disease, COVID-19 still affects health after recovery. The late symptoms of SARS-CoV-2 (also called Long-COVID) are diverse (53). The most common are exhaustion, difficulties in breathing, and muscle weakness followed by sleep disorders as well as cognitive disorders and depression, but also a significant increase in Diabetes type I is reported, especially in children (54, 55). How frequent symptoms occur and how long patients are affected strongly differ. Women are somewhat more affected (56). The late symptoms of COVID-19 are very nonspecific and sometimes difficult to assess (57, 58). Previous studies primarily cover affected persons with severe progressions and indicate a correlation of Long-COVID to the specific titer of IgM and IgG3 (53, 59, 60). An evaluation of mild progression with corresponding symptoms at the beginning of the disease as well as a follow-up of the corresponding patients to Long-COVID does not yet exist. A study on the correlation of direct and Long-COVID syndrome with comparative antibody concentrations in patients is also not yet available.

In summary, it is not yet clear, how long the humoral immunity lasts after a SARS-CoV-2 infection or vaccination. Another open question is, whether the existing serological tests and their different detection principles and used antigens reflect the kinetics of individual immune responses upon infection and mild progression in a comparable way. Also, no comprehensive evaluation of serological methods upon recent WHO standardization of the tests, a uniform cutoff, and the correlation to the neutralizing property of the respective immune-titer is available in this mildly affected cohort so far. This ongoing debate on the suitability of serological tests and their correlation to neutralizing assays is well summarized in the recent publications by Castillo-Olivares et al. and Lippi et al. (50, 61)

To investigate these questions, we compared the antibody response of 13 COVID-19 patients (confirmed by qRT-PCR) displaying mild COVID-19 symptoms up to 455 days post-infection to those of eight healthy control individuals (one unvaccinated, six fully vaccinated and one vaccinated post-COVID-19 infection). Antibody response after infection or vaccination, respectively, was determined simultaneously using four different quantitative immunoassays (detecting either antibodies against the S protein or the RBD) and one quantitative surrogate immunoassay to determine neutralizing antibodies. Quantitative surrogate immunoassay of neutralizing antibodies have been demonstrated to correlate with direct live cell-based neutralization assays (49, 62–64). In contrast to cell-based neutralization assays, surrogate immunoassay of neutralizing antibodies can be easily performed in all laboratories without the need for high biosafety level 3 (65, 66). Furthermore, a qualitative immunoassay detecting antibodies against the N protein was applied to distinguish between virus infection and vaccination.

## Material and methods

### Patient samples

In this retrospective study, all serum samples sent to our laboratory for SARS-CoV-2-IgG determination between March 2020 and June 2021 from participants with a positive result of SARS−CoV-2 RT-PCR in a nasopharyngeal swab between March and April 2021 (at least 10 days before serum collection) were considered for analysis (n = 169). At the time of the start of the study (March 2020), VOC of SARS-CoV-2 were not present in Germany and no routine molecular diagnostics to differentiate among viral subtypes was available at this time. For this no further information is reported on the genetic background of SARS-CoV-2 of the participants. Information about clinical symptoms and the day of onset of symptoms and on repeated examination of participants in the course of the study were obtained.by respective medical doctors. Physician were provided a standardized questionnaire to check and report on appearance, frequency and intensity of symptoms. Participants that experienced problems on vaccination (beside fatigue, irritation/painful injection site for 2-3 days) were also excluded from this study. Participants with hospital treatment for COVID-19 (n = 38) and participants in whom clinical information could not be obtained (n = 72) have been excluded from the analysis. All together 59 follow-up samples from 13 participants fulfilling the clinical diagnostic criteria for SARS-CoV-2 remained for further analysis (67). Additionally, serum samples of six healthy fully vaccinated individuals (3x Comirnaty^®^, BioNTech/Pfizer; 3x Spikevax^®^, Moderna) and one post-COVID-19 vaccinated participant with Comirnaty^®^, BioNTech/Pfizer were included in the analysis. Both vaccines used are RNA-based. Pfizer/BioNTech (BNT16b2) is administered intramuscularly 30 μg per dose (0.3 ml) on an injection dose interval of 21 days, second dose. Moderna (mRNA-1273) is administered intramuscularly 100 μg per dose (0.5 ml) on an injection dose interval of 28 days, second dose. Further characteristics on efficacy and effectiveness against SARS-CoV-2 of these vaccines are summarized in Fiolet et al. (68) Samples of participants were frozen after routine analysis was finalized and stored at -80°C until respective measurements.

### Assays and instruments

One qualitative and four quantitative immunoassays were applied to determine SARS-CoV-2 antibodies. The SARS-CoV-2 UTAB FS (RBD-based antigen, DiaSys Diagnostic Systems GmbH Holzheim, Germany) was performed on the Cobas 8000^©^ c502 (Roche Diagnostics, Mannheim, Germany). The Elecsys^®^ Anti-SARS-CoV-2 (N-based) and Elecsys^®^ Anti-SARS-CoV-2-S (RBD-based) were performed both on the Cobas 8000^©^ e601 (Roche Diagnostics, Mannheim, Germany). The Liaison^®^ SARS-CoV-2 TrimericS IgG was performed using the Liaison^®^ XL (S-based; DiaSorin, Dietzenbach, Germany). The Anti-SARS-CoV-2-QuantiVac ELISA IgG (S1 antigen-based, EUROIMMUN, Luebeck, Germany) was conducted according to the manufacturer’s instructions and data were recorded using a Sunrise™ absorbance microplate reader (Tecan Group, Maennedorf, Switzerland).

One quantitative surrogate immunoassay was applied to determine the SARS-CoV-2 neutralizing antibodies. The SARS-CoV-2 Neutralization Antibody Assay (TECOmedical AG, Sissach, Switzerland) was conducted according to the manufacturer’s instructions and data were assessed using a Sunrise™ absorbance microplate reader (Tecan Group, Maennedorf, Switzerland).

All quantitative immunoassays were calibrated to the WHO International Standard for anti-SARS-CoV-2 immunoglobulin (human) (NIBSC Code 20-136) (69) and results were evaluated according to **Table 1**. All measurements were performed in parallel after thawing and careful homogenization of samples to ensure a comparable setting on each instrument and assay.

**Table 1:** Result interpretation.

Manufacturer:	DiaSorin	DiaSys	EUROIMMUN	ROCHE		TECO
**Assay name:**	LIAISON^®^ SARS-CoV-2 TrimercS IgG	SARS-CoV-2 UTAB FS	Anti-SARS-CoV-2 QuantiVac ELISA (IgG)	Elecsys^®^ Anti-SARS-CoV-2	Elecsys^®^ Anti-SARS-CoV-2 S	SARS-CoV-2 Neutralizing Antibody Assay
**Antigen**	Spike Trimer	Spike RBD	Spike S1 (incl. RBD)	Nucleocapsid	Spike RBD	Spike RBD
**specific units:**	BAU/ml	BAU/ml	BAU/ml	COI	U/ml*	IU/ml
**negative/not reactive:**	< 33.8	≤ 30	< 25.6	< 1.0	< 0.8	< 20.00
**intermediate:**	–	–	25.6-35.2	–	–	–
**positive/reactive:**	≥ 33.8	> 30	> 35.2	≥ 1.0	≥ 0.8	≥ 20.00

*Manufacturer-specific U/ml are considered as equivalent to BAU/ml, based on the manufacturer’s applicable documents (70).

### Statistical analysis

Calculation and statistical analyses were performed using XLSTAT^®^ software, version 2016.06.35661 (NY, USA), following the principles of C24A3E-Statistical Quality Control for Quantitative Measurement Procedures: Principles and Definitions; Approved Guideline–Third Edition. MedCalc^®^ Version 18.10.2 – 64-bit (MedCalc Software Ltd, Belgium MedCalc Software bvba, Ostend, Belgium; http://www.medcalc.org; 2018) was used for Passing & Bablok regression by its particular function “Scatter diagram & regression line”.

### Institutional review board statement

The retrospective evaluation was exclusively performed on pre-existing patient samples obtained after routine analysis was completed. All the leftover samples were completely anonymized and de-identified. The study has been approved by the local ethics committee (Ärztekammer Sachsen-Anhalt, No. 100/21) and is registered by DRKS-ID DRKS00028039. The research complied with the World Medical Association Declaration of Helsinki regarding the ethical conduct of research (71).

## Results

### Characterization of participants and sero-assay performance of SARS-CoV-2 recovered participants

Three to five serum samples from 13 participants collected between day 11 and 455 after the onset of symptoms were analyzed to study the antibody levels longitudinally post COVID-19 infection. Clinical data from three male and ten female participants aged between 20 and 61 (mean 50.5) were obtained (**Table 2**). Seven participants had contact to an RT-PCR confirmed COVID-19 patient and all participants had only mild symptoms such as fever, cough, general weakness/fatigue, headache, myalgia, sore throat, coryza, dyspnea, anosmia or ageusia (mean six of ten symptoms). None of the participants had a chronic respiratory or coronary disease, adiposity, or diabetes. One participant was taking immunosuppressive drugs due to rheumatoid arthritis. In addition to the detection of antibody titers, symptoms of long-term consequences of COVID-19 disease were recorded for the corresponding participants after the infection had subsided (Long-COVID symptoms).

**Table 2:** Medical background of participants recovered from mild COVID-19 (No. 1 -13) and of vaccinated participants (No. 14 - 20).

	Participant					COVID-19 symptoms																		drugs	Long-COVID
COVID-19 (COV) vs. vaccinated (VAC)	No.	Age	Sex	RT-PCR positive after first symptoms (days)	contact to COVID-19	fever (>38°C)	cough		weakness/fatigue		headache		myalgia		sore throat		coryza		dyspnea		anosmia		ageusia	Immuno-suppressive	
COV	1	36	f	4	–	+	+		+		+		+		+		–		–		+		+	–	–
COV	2	57	f	1	+	+	–		+		+		+		–		–		–		+		+	–	–
COV	3	58	m	2	–	+	–		+		+		+		–		+		–		+		+	–	–
COV	4	61	f	4	+	–	–		+		+		+		–		–		+		–		–	–	+
COV	5	53	f	4	+	–	+		+		+		+		–		–		+		+		+	–	+
COV	6	20	f	4	–	–	–		–		+		+		–		–		–		+		+	–	n.d.a.
COV	7	55	f	2	–	+	–		+		–		–		+		–		–		+		+	–	n.d.a.
COV	8	56	m	2	+	+	+		+		+		+		–		–		+		+		+	–	+
COV	9	55	f	4	–	+	+		+		+		+		+		–		+		+		+	–	+
COV	10	55	f	4	–	–	–		+		–		–		+		–		+		+		+	+	n.d.a.
COV	11	56	m	5	+	+	–		+		–		+		+		–		+		–		–	–	n.d.a.
COV	12	46	f	4	+	–	–		+		–		–		+		+		–		+		+	–	+
COV	13	49	f	5	+	+	+		+		+		+		–		–		–		–		–	–	+
VAC	14	50	m	n.d.a.	–	n.d.a.																		–	n.d.a.
VAC	15	56	f	n.d.a.	–	n.d.a.																		–	n.d.a.
VAC	16	62	m	n.d.a.	–	n.d.a.																		–	n.d.a.
VAC	17	57	f	n.d.a.	–	n.d.a.																		–	n.d.a.
VAC	18	52	f	n.d.a.	–	n.d.a.																		–	n.d.a.
VAC	19	37	f	n.d.a.	–	n.d.a.																		–	n.d.a.
COV/VAC	20	46	f	n.d.a.	+	+		–		+		+		+		+		+		–		+		+	–	+

Chronic diseases such as respiratory diseases, coronary diseases, diabetes or adiposity were not reported. Participants that experienced problems on vaccination (beside fatigue, irritation/painful injection site for 2-3 days) were excluded from this study. For a more detailed description on Long-COVID symptoms please refer to **Supplement Table 1**. Participants that did not show symptoms (vaccinated group) or did not agree on disclosure of specific symptoms on Long-COVID are indicated (n.d.a.).

**Figure 1:**
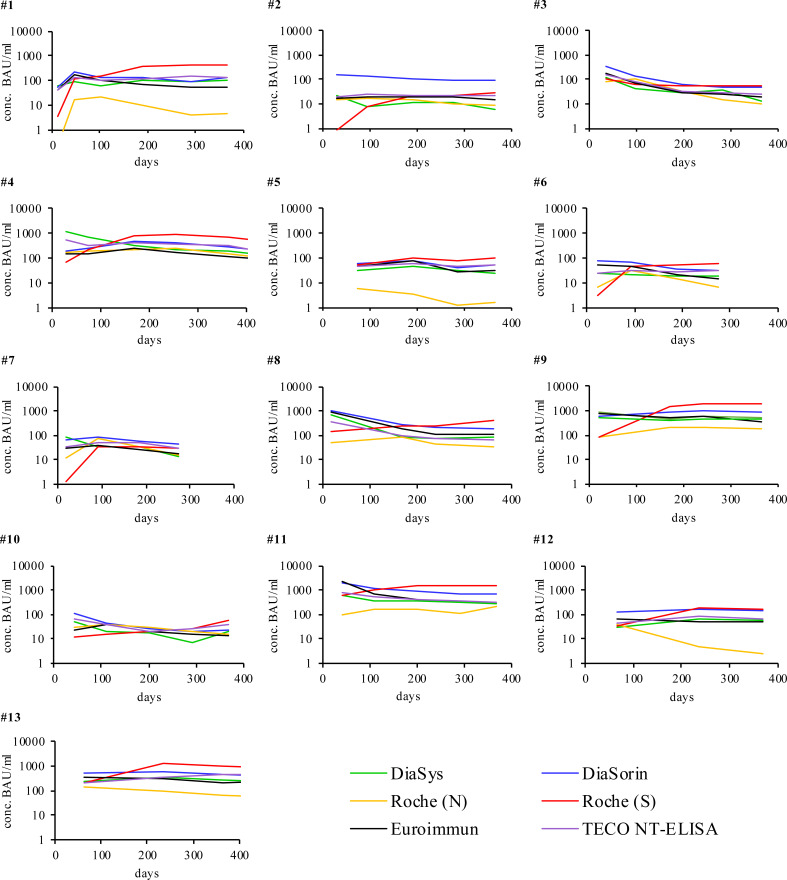
Individual humoral antibody immune response of participants recovered from COVID-19 monitored over respective days with different immuno-assays. Signals were measured in BAU/ml (y-axis) except for Roche (N) in cut-off-index (COI). The respective cut off values of the different assays are reported in **Table 1**. X-axis represents duration of evaluation in days. Participants 6 and 7 stopped after 270 days due to the first vaccination. For a more detailed analysis of the participants please also refer to **Supplement Table 3**.

SARS-CoV-2 antibody levels in COVID-19 recovered participant’ samples were measured with six different immuno-assays simultaneously (five sero-assays and one quantitative surrogate immunoassay to determine neutralizing antibodies). All participants developed antibodies against SARS-CoV-2 though in quite different levels (**Figure 1**). In general, over time a steady decrease of detectable antibodies always resulted in a persisting, stable condition up to one year post-infection (**Supplement Figure 4** and **Supplement Table 3** and **4**). The specific antibody levels of the observed participants showed significant differences in scale. In particular four participants (2, 6, 7, and 10) developed only low antibody quantities sometimes near their respective assay positive cut-off (20-50 Binding Antibody Units (BAU/ml)). Two participants (5 and 12) developed low to mid amounts of antibodies (up to 200 BAU/ml) while four participants (1, 3, 4, and 8) showed high amounts (up to 1000 BAU/ml). Very high amounts (>1000 BAU/ml were found in three participants (9, 11, 13). For all participants, any detected antibodies reacted neutralizing in the TECO-neutralization assay preventing recombinant viral spike-RBD from binding to ACE2.

### Individual immune response of vaccinated participants

**Figure 2:**
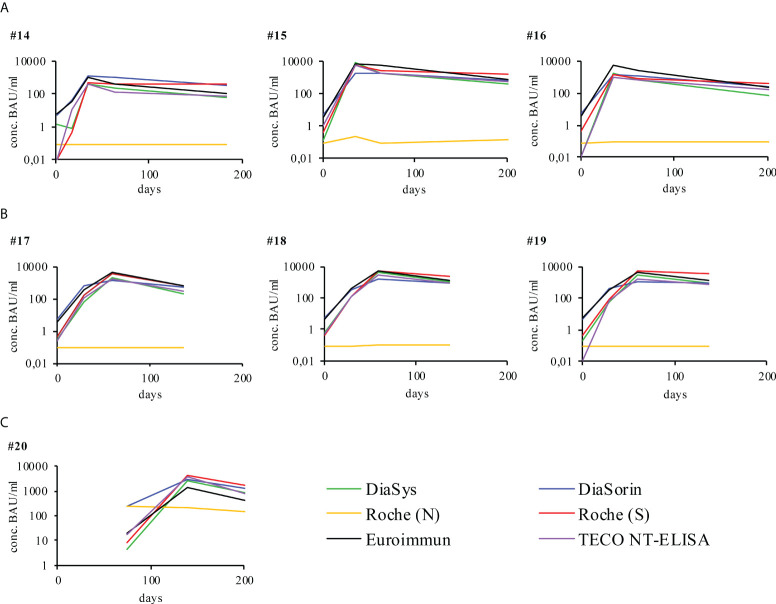
Individual humoral antibody immune response of vaccinated participants monitored over respective days post vaccination with different immuno-assays. All signals were measured in BAU/ml as indicated by respective manufacturer (y-axis), except for Roche (N), the latter was measured in cut-off-index (COI). The respective cut off values of the different assays are reported in **Table 1**. X-axis represents duration of evaluation in days. Participants were vaccinated with either **(A)** Comirnaty®, BioNTech/Pfizer or **(B)** Spikevax®, Moderna. Participant 20 was vaccinated with Comirnaty® after COVID-19 recovery, represented by a later start of vaccination specific data **(C)**. For a more detailed analysis on of the participants please also refer to **Supplement Table 3**.

**Figure 3:**
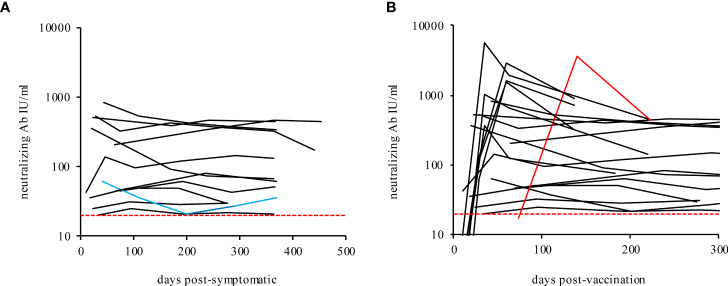
Neutralizing antibodies in recovered mild COVID-19 participants **(A)** and vaccinated participants **(B)** over time. The blue line shows the immune-suppressed participant 10. The red line shows participant 20 (post-COVID-19 and fully vaccinated with Comirnaty®, BioNTech/Pfizer). The dashed lines indicate the TECO assay positive cut-off (20 IU/ml).

Seven participants fully vaccinated with either Comirnaty^®^ (BioNTech/Pfizer) or Spikevax^®^ (Moderna) were measured accordingly (**Figure 2**). In contrast to recovered participants, the vaccination resulted in a higher overall production of anti-Spike-protein antibodies while anti-Nucleocapsid-antibodies were not detectable. The only fully vaccinated participant that showed detectable anti-N-antibodies was recovered from prior COVID-19 (**Figure 2C**, participant 20). Although antibody titers rapidly increased to their maximum they decreased subsequently. However, they never dropped below respective assay cut-offs but rather seemed to stabilize. The observed minimal antibody levels of vaccinated participants remain at an overall higher level, compared to the minimal level of infected participant (overall median minimal antibody level infected participans 62.4 IU/ml; overall median minimal antibody level vaccinated participants 446.5 IU/ml, **Supplement Figure 5A**). The maximal value is reported in relation to the initial antibody value (145.6 IU/ml infected participants, 1615.4 IU/ml vaccinated participants, **Supplement Figure 5B**). The maximal decrease was calculated in respect to this maximal observed level of antibody concentration. The observed timepoints of maximal increase as well as the kinetics of decrease do strongly vary among participants. Further information on initial, maximum, minimum and mean antibody levels, detected in vaccinated participants and infected participants is summerized in **Supplement Table 3**. A comparison of the decline rates of antibody levels, detected in COVID-19 patients and vaccinated participants is given in **Supplement Table 4** and **Supplement Figure 4**. Antibody levels of TECO NT method (IU/mL) were calculated in %, setting the highest value to 100%. Concentration values of the measuring times before highest concentration were not considered. On the contrary to vaccinated participants, concentrations of the COVID-19 patients are lower at the beginning but they remain constant in general during the time. The two regression lines clearly differ (slopes p = 0.009503, intercepts p = 0.006324), indicating the rapid decrease of vaccinated participants in contrast to that of COVID-19 recovered participants. For all participants the neutralization assay also showed that those antibodies have an inhibiting effect (**Figure 3**).

### Correlation of neutralizing antibodies in recovered mild COVID-19 as well as vaccinated participants

**Figure 4:**
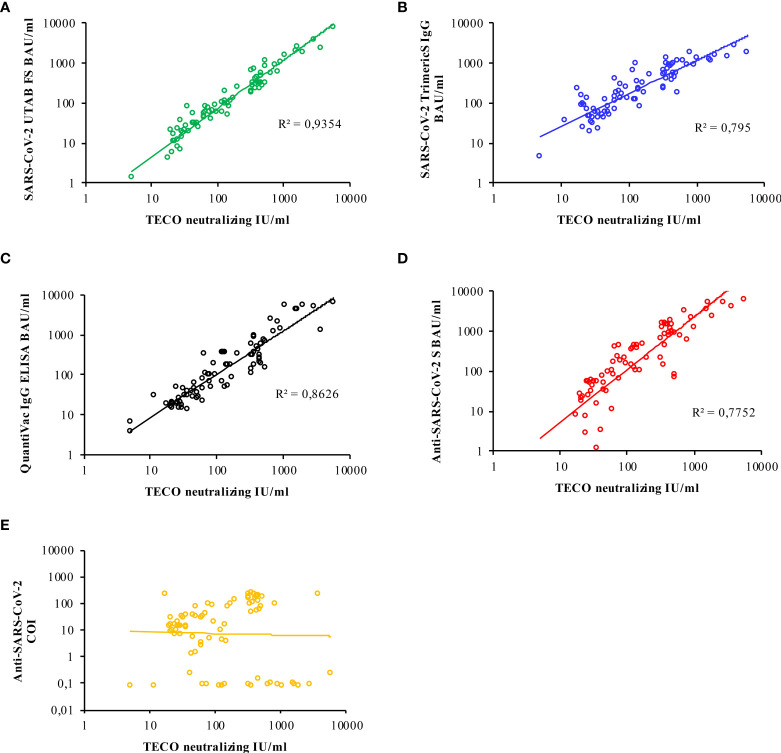
Logarithmic correlation for each immuno-assay **(A–E)**, compared to neutralizing antibody titer measured with the TECO-ELISA. Results for Roche N-Test **(E)** are only semi-quantitative and reported as cut-off-index (COI); due to observed kind of correlation of Roche N, no R2 is indicated. A total of 83 samples (COVID-19 patients and vaccinated individuals) was measured. Additional evaluation of corresponding correlations by Passing & Bablok / Spearman coefficient is given in **Supplement Figure 1B**.

According to the manufacturer’s instructions, all assays except for the Elecsys^®^ Anti-SARS-CoV-2 (N-based) are calibrated to the first WHO International Standard. Thus, a direct comparison of obtained results with those assays is largely possible. The result of each applied assay was plotted against respective values obtained from TECO neutralization assay to assess correlation (**Figure 4**). However, the closest correlation was found to SARS-CoV-2-UTAB FS from DiaSys whereas the highest deviation occurred with Elecsys^®^ Anti-SARS-CoV-2 (S-based) assay from Roche. Since the Elecsys^®^ Anti-SARS-CoV-2 (N-based) assay was a semi-quantitative assay, a confident correlation could not be carried out.

**Figure 5:**
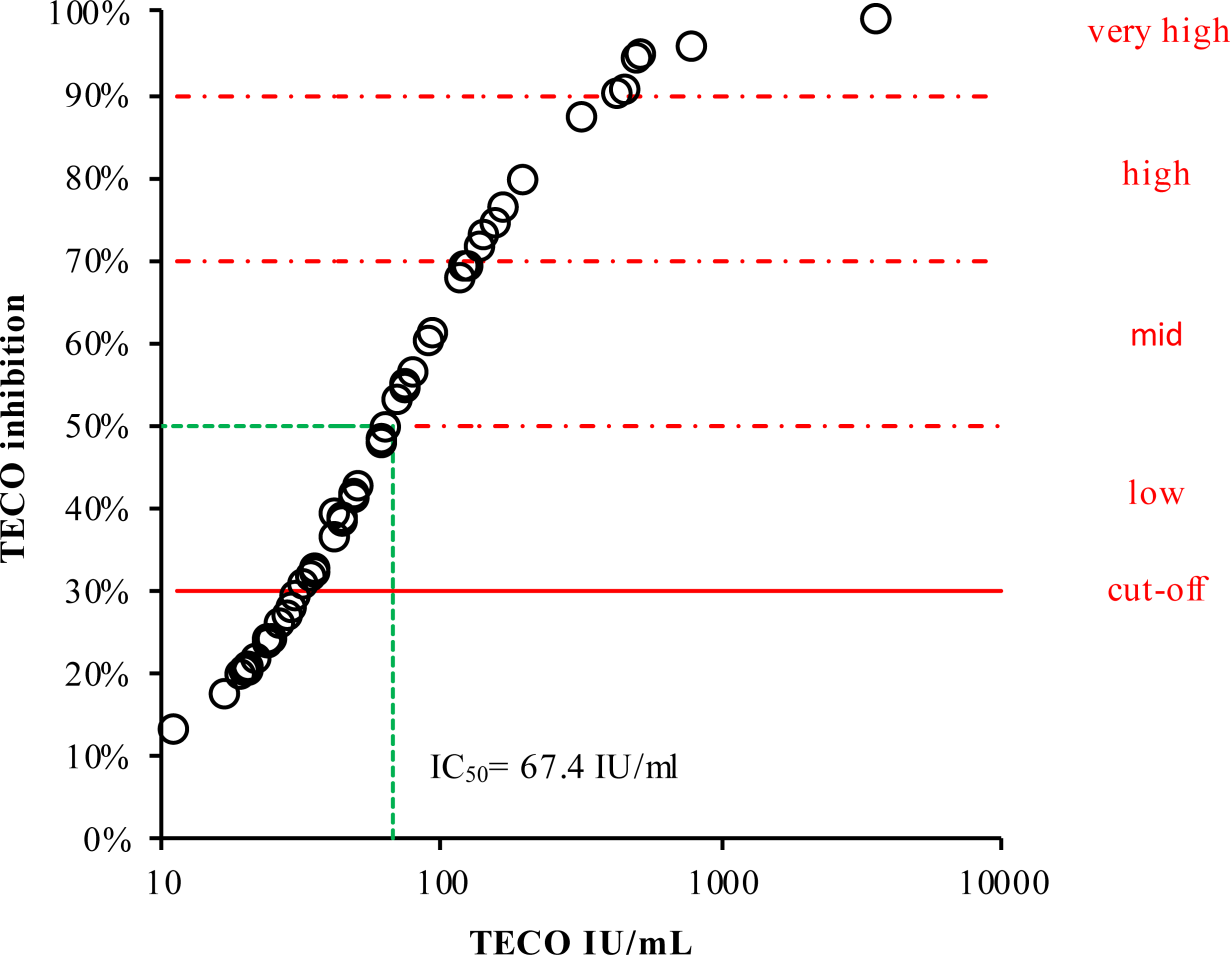
Inhibition curve of neutralizing antibodies divided into inhibition efficacy groups dependent on respective measured antibody concentration. The cut-off is given as a continuous red line. For better visualization and estimation, the neutralizing potential was marked by gradual inhibition areas (dashed red lines). For classification of antibody titers (in %), according to their inhibition potency (IU/mL, derived from TECO neutralization assay), please also refer to **Table 3**. Green dashed line represents half-maximal inhibition (50%), corresponding to 67.4 IU/ml.

Furthermore, the results from participants’ samples obtained with the TECO neutralization assay (IU/ml) were calculated into inhibition values and were plotted against the respective antibody titers (**Figure 5**). This plot resulted in a typical sigmoidal saturation curve with a linear behavior between 33.16 to 170.21 IU/ml (30% to 76% inhibition, respectively) with R^2^ = 0.9985 (**Supplement Figure 2**). Exceeding 170.21 IU/ml, the curve flattened almost reaching saturation. With the help of this curve, IU/ml values of serological testes could be transferred to the percentage of inhibition (**Table 3**). Based on this conversion we roughly divided and classified inhibition efficacy groups concerning their inhibition potency, revealing a half-maximal inhibition at 67.4 IU/ml.

**Table 3:** Classification of antibody titers according to their inhibition potency derived from TECO neutralization assay.

	inhibition %	neutralizing IU/ml
**negative**	≤ 30	< 33.16
**low**	30 - 49	≥ 33.16 – 65.86
**mid**	50 - 69	> 65.86 – 126.05
**high**	70 - 89	> 126.05 – 434.43
**very high**	≥ 90	> 434.43

## Discussion

In this study, we did directly compare all so far utilized methodical principles (ELISA, enzyme-linked immunosorbent assay; CLIA, chemiluminescence immunoassays, PETIA, particle-enhanced turbidimetric immunoassay) and different bound antigens for quantitative detection of SARS-CoV-2 specific antibodies (N-, S1- and RBD-antigens). Only assay systems suitable for high throughput platforms of the clinical laboratory were evaluated. Qualitative “lateral flow” kind of assays were not considered for this evaluation.

To assess the suitability of all evaluated test systems concerning the mentioned heterogeneity of antibody dynamics and the binding to the antigen of the test system, 13 participants were continuously measured over a period up to 455 days, directly after the onset of SARS-CoV-2 specific symptoms. A clear limitation of this study is the small sample size, based on the early start of the study in Germany in March 2020 and also in the willingness of participants to take part in a longitudinal study of 455 days. Motivation of participants to continue also was challenging in the course of the study due to the upcoming controversial political debates on SARS-CoV-2. Consequently, the clear focus of the present work is the long-term monitoring and direct comparison of all principal methods of quantifying the immune response of patients upon infection with SARS-CoV-2 or vaccination by routine high throughput serological assays.

Only participants with a SARS-CoV-2 infection that was confirmed by RT-PCR were considered, as was one SARS-CoV-2 positive participant with a suppressed immune system (**Figure 1**, participant No. 10). Except for the Roche S test, all other systems similarly map the dynamics of individual participants from the onset to the continuous drop of antibodies within 455 days (**Figure 1**). For all participants (with exception of participants No. 3, 5), Roche S shows a lower starting signal and a comparable but slightly increased dynamic in long time monitoring (except participants No. 8 and 10, which reveal a constant increase over time). Compared to all other assay systems, Roche (S- and N-) are working with a significantly lower cutoff (0.8 U/ml and 1 COI compared to 20-35 Binding Antibody Units (BAU)/ml, see also **Table 1**). The overall observed low initial signal, as well as some increase at higher values, maybe due to the mode of BAU/ml-standardization of Roche S, especially at the cutoff, affecting the dynamics of the calibration on the whole analytical range. As Roche utilized an RBD-antigen same as other manufacturers, and also detects multiple isotypes (IgA, IgM, and IgG, **Supplement Table 2**), this observed effect probably is not associated to the different binding properties of antibody isotypes to the chosen RBD-antigen of the Roche S assay.

In the light of the recent WHO standardization of all evaluated serological tests, the difference in the absolute signal of all tests is striking (all reported in BAU/ml, **Figure 1** and **Supplement Figure 1**, **3**). This especially ascribes to **Supplement Figure 3** on the overview of the linear correlation among all evaluated assays. Also, all manufacturers report to be traceable to the material of WHO (WHO/BS/2020.2403; NIBSC code 20/136) (69) and standardized to BAU/ml, all assays show remarkable differences by direct linear comparison and recovery of samples. The observed variation among tests, even after correlation to the WHO standard is in accordance with resent work by Perkmann et al. (72) The difference, in particular, applies to the onset of the immune response. A different recognition of the antibody subclasses by the respective tests may explain this finding. Also, the composition of the WHO standard itself, which may not sufficiently reflect variability and dynamics in its immunoglobulin composition may contribute to the observed effect.

The N-based test from Roche was used to evaluate and confirm deviations in the detection between S/RBD and N- as described in the literature (**Figure 1**, participants No. 1-13) and also to check the reactivity after vaccination with S1- or RBD-based RNA vaccine (**Figure 2**, participants No. 14-20) (5, 6, 20–24). Striking significantly different kinetic progressions can be observed by the use of the N-test of Roche in **Figure 1**, in particular participants No. 5, 12, and 13 with a significant drop, compared to the other test systems. Antibodies to the viral N-protein decline faster than those to the receptor-binding domain or the entire spike protein (24). The reason for the observed faster decline in some participants remains unclear. In the group of vaccinated participants (**Figure 2**), participant No. 20 attributes an exceptional role. This participant, despite positive PCR and also positive on N-antibody sero-status upon subsided infection, did not form S-protein derived antibodies above the limit of detection. Strikingly S-protein derived antibodies in participant No. 20 first did emerge after vaccination (**Figure 2C**, participant 20). This participant may have been affected by a very rapidly subsiding infection in which larger amounts of viral proteins were released by virus degradation and lysis. As the N-protein by far is the most abundant protein in SARS-CoV-2, immune reactivity directed against N-protein of SARS-CoV-2 is preferred (20, 24). Due to a rapid elimination of the virus and its fragments, only marginal reactivity of S-/RBD-specific antibodies may have occurred. Also a preceding infection with another human pathogenic coronavirus (HCoV), leading to a de-sensibilization of the S-/RBD-derived immune response may have biased the observed low values of S-/RBD-derived antibodies (73, 74). As this is a single case observation, interpretation needs to be handled with care and remains unsolved.

As expected, the N-antigen is increased in participant 20 due to a previous infection. For participants 14-19 there is no increase with the Roche N test. The reactivity and dynamics after vaccination show similar shape and height depending on the starting values and patient-specific speed of the immune response (**Figure 2**) by all sero-assays. Also, the participant with a suppressed immune system (No. 10) shows comparable dynamics in response to the infection in all tests. Probably due to administered immuno-suppressive substances, this participant revealed an overall weak immune response and also a rapid decline. As only one participant of this study was presenting with immune suppressive medication, this observation needs to be considered with care. Already after 200 days, post-infection neutralization potential was marginal. Comparing the vaccination derived immune-response with the response initiated by a SARS-CoV-2 infection, striking different dynamics are evident: While infection-derived antibody titers rise to 1000 BAU and stay constant over 455 days, vaccination-derived ones substantially rise to 5000 - 10000 BAU/ml but also drop fast in a short time (**Supplement Table 3**, **4** and **Supplement Figure 4**). The observed fast decline rates of vaccinated participants on average decreased within 100 days post vaccination to the titers that infected participants do reach after 455 days - and continue to decrease. In this context it is important to note, that, during the time of the study, the observed drop in minimal antibody levels in vaccinated participants remain on a significant higher level, compared to the minimal level of infected participant (overall median minimal antibody level infected participants 62.4 IU/ml; overall median minimal antibody level vaccinated participants 446.5 IU/ml, **Supplement Figure 5**). In addition, most SARS-CoV-2 infected participants already revealed the maximal antibody titer at the initial time point/quantification of the study (**Supplement Table 3**). This probably is due to the well-defined time of vaccination and the blood sampling at an early stage of onset of the immune response of the vaccinated group, compared to the variable and sometimes quite late time point of first presentation and blood sampling of the infected participants. This is especially the case since in this study only mild courses of infection and symptoms were considered for this study. For this reason, onset of the immune response and isotype switch of antibodies may have already occurred at the first time point of sampling. As this study does focus on the overall kinetics of the antibody titer and their reactivity towards different methods of quantification of SARS-CoV-2 derived sero-titers, the initial time point of infected participants probably has minor impact to this study. However, a putative effect of antibody conversion among different methods was already addressed in a previous study by Spaeth at al (16).

A subsided infection with SARS-CoV-2 or a corresponding vaccination provides a certain protection against re-infection with SARS-CoV-2. To what extent, duration and to which threshold level of antibodies the acquired immunity is sufficient to gain a protective immune-response currently cannot conclusively be answered by so far literature (75–79). Methodologically, the detection of antibodies that block entry of the virus into the cell, primarily in the area of the S1/RBD structure of SARS-CoV-2, represents the gold standard for quantifying immune protection (SARS-CoV-2 virus neutralization tests or neutralization surrogate tests). For most serological methods, this has not yet been comprehensively and directly compared. In this study, the determined values of the individual serological tests were set in reference to analogous measurements with a quantitative surrogate immunoassay (TECOmedical AG) to reflect the effective immunological protection of SARS-CoV-2 neutralizing antibodies (**Figure 3**, reported in IU/ml). As shown in **Figure 3A** and **Supplement Table 3**, the neutralizing antibody activity in all recovered participants remain stable throughout the study period. In contrast, the protective immune response after vaccination does reveal an exaggerated increase, followed by a rapid drop within the period of measurement (**Figure 3B** and **Supplement Table 3**, **4** and **Supplement Figure 4**), supporting a recent publication about a less sustainable immune protection by RNA-vaccination (80). Interestingly the neutralizing titer, measured by the surrogate virus neutralization test declines over time in some participants, while anti-RBD or anti-S titers, measured by serological assays, seem to remain constant. Our data confirm similar observations previously pointed out by L’Huillier et al. (81) The observed effect could be ascribable to potential biased results for the anti-RBD/anti-S measurement of serological assays. Indeed, some of the assays employed in the present work do determine total Ig and not only IgG (see also **Supplement Table 2**). Furthermore, the anti-RBD or anti-S assays results are more affected by higher-affinity antibodies. Consequently, the anti-RBD or anti-S measuring immunoassays could generate an increased signal. This in turn is indicating higher antibody concentrations, what actually would reflect antibody affinity maturation over time more than the concentrations themselves. The latter actually should maintain stable or even decreases during time, as already supposed by L’Huillier and colleagues (81).

To directly compare whether and to which extent the serological tests reflect virus-neutralizing protection, the TECOmedical values were correlated to the individual serological tests (**Figure 4** and **Supplement Figure 1**). Besides Roche N Test, all tests revealed a good agreement with the neutralization surrogate test. Interestingly the DiaSys SARS-CoV-2 PETIA test exhibits an excellent correlation (R^2^ = 0,92) as well as a low degree of scattering, compared to all other tests, showing an R^2^ in between 0,64 and 0,75 (**Supplement Figure 1A**). However, please note, that due to the principle of the surrogate neutralization test on generating kinetics of immune-inhibition this can only be compared to serological test based on S- or RBD- antigens and to tests, well-standardized to BAU/ml. For this, the N antigen-based test by Roche cannot be directly compared to neutralization inhibition testing *per se*.

Although neutralization tests are seen as the gold standard for detecting the neutralizing potential of SARS-CoV-2 specific antibodies, there is currently no reliable classification or value assignment available (see also resent review on this debate by Lippi et al.) (61). In a further report, Castillo-Olivares et al. compared a variety of commercial and non-commercial sero-tests and neutralization assays (ranging from lateral flow test, S-/N-based ELISAs, Roche N and S ECLIA, multiplexed particle flow cytometry assay, multiplex antigen semi-automated immuno-blotting pseudo-typed microneutralization test and electroporation-dependent neutralization assay) in mild, moderate and severe infections. This short-term study (up to 5 month) by Castillo-Olivares et al. indicated, based on a pseudo-type virus and standardization into IU/ml or BAU, that overall, severe COVID-19 patients showed higher levels of SARS-CoV-2-specific neutralizing antibodies (average 1029 IU/ml) compared to those observed in seropositive mild or asymptomatic infections (379 IU/ml). Clinical severity in the study of Castillo-Olivares et al. was tightly correlated with neutralization and RBD/S antibodies. In addition, there was a positive correlation between severity, N-antibody assays and intracellular virus neutralization (50). Due to good overall accordance with the work of Castillo-Olivares et al. and the good agreement of all S-/RBD-based serological tests observed in our long-term study, a classification based on the shape of the inhibitions curve was derived in this work in addition to the cutoff given by the manufacturers of commercial high throughput routine assays (20 IU/ml). To this end, the values of serological testes could be converted to the percentage of inhibition of the TECO neutralization assay, revealing a half-maximal inhibition at 67.4 IU/ml (**Table 3** and **Figure 5**). Please note that also it is possible to transfer IU/ml to the percentage of inhibition, an inhibitory saturation curve only is possible to be used to a limited extent for all tests, even if these tests are standardized to BAU/ml. Most tests are structured very differently utilizing the target antigen (e.g., based on spike protein, only RBD spike, spike trimer, etc.), its way of production (e.g., recombinant in bacteria or eukaryotic cells), Lot to Lot variation, and purity of the antigens. Also, in regard to VOC, the kinetics of neutralization probably are different. This especially may be due to the recent omicron variants of SARS-CoV-2, characterized by several variations within the RBD area of the viral spike structure (45, 82–84). To obtain solid and robust conversion factors, long-time surveys on different lots and cohorts of patients are necessary. The data provided here clearly point out that a common conversion is achievable on serological and neutralization tests. However, the observed test variations and new SARS-CoV-2 variants demonstrate that up to now it is difficult to define a cut off value for immune protection as suggested recently. A randomized efficacy trial of the ChAdOx1 nCoV-19 (AZD1222) vaccine in the United Kingdom analyzed the antibody levels associated with protection against SARS-CoV-2 and did show approximate 80% efficacy of a vaccine at 26 IU/ml. Binding and neutralizing antibodies at 28 days after the second dose in this study were measured in infected and noninfected vaccine recipients. Anti-SARS-CoV-2 Spike and RBD IgG were measured by a multiplex immunoassay on the MSD platform (85). A further, very recent study by Cantoni et al. indicated that, using an estimated threshold of 50% protection (corresponding to 54 IU/ml as also indicated by Khoury et al. (39)), that most asymptomatic and mild cases of SARS-CoV-2 did not produce titers above this cut off (49). The work by Feng et al., Cantoni et al. and our own work implies, that an overall correlation of sero-tests and neutralizing assays appears to be possible on the respectively used methods. The strong methodical assay heterogeneity among these studies, the variety of used analyzers and platforms, the sample material used for correlation (infected vs. vaccinated, varying VOC background as well as individual sero-conversion and Ig-isotypes), and the challenge of traceability to an international standard still seem to limit a universally valid transfer up to now. Considering also the high structural dynamics of the Spike-structure of SARS-CoV-2 itself and the derived consequence in a varying individual immune response could also impede a clear conversion and a defined cut off (21). This topic may also need guidance of national and international organizations on standardization of SARS-CoV-2.

Many reports differ regarding variation or changes in titer levels in mild vs. severe cases of COVID-19 (7–10, 28–38). For this, the clear characterization of participants and the assessment of symptoms during the progression of the infection was an important aspect of this study. Participants were included in this study, when the respective RT-PCT result did confirm a SARS-CoV-2 infection. Besides age and sex, COVID-19 symptoms, further medication, and chronic diseases were reported (**Table 2**). During the progress of the study also Long-COVID symptoms were assessed in addition (**Supplement Table 1**). The kind and frequency of symptoms in this cohort of mild progression (**Supplement Table 1**) are in good agreement with recently published studies (57, 58), also the distribution of Long-COVID is in line with a very recent work by Huang et al. (59) The number of symptoms of this cohort during the onset of infection or Long-COVID, however, seem not to be associated with the intensity or dynamics of the immune response in all participants of this study. In this context it is important to note, that the small size of SARS-CoV-2 infected participants (3 males and 10 females) are limited in their statistical power in interpretation of the observed relation of symptoms, sex, age or Long-COVID. The presented data primarily serve for a robust characterization of the presented participants.

Taken together, the data presented here show that the immune responses over 455 days after a mild symptomatic SARS-CoV-2 infection is very individual and although there is a moderate decline throughout the study period the antibody levels of all COVID-19 patients reach a stable plateau, independent whether weak or strongly seropositive. All participants exhibit neutralizing antibodies in the period of the survey. Also, a good overall correlation to the total SARS-CoV-2 antibody content of all assays can be observed. Antibody stability upon infection is much more pronounced compared to a vaccination-derived immune response. The observed dynamics of the immune response after infection also do not seem to show a relation to the number of symptoms, differences in sex, or any age-related dependency or Long-COVID. Overall, all evaluated tests reveal comparable dynamics within the 455 days of data collection. Roche S in particular has chosen a different cutoff and also has the strongest deviations from the other tests. All serological tests can be compared well against a surrogate neutralization test. An estimation of the neutralization potential derived from the serological tests also is possible on the evaluated assays and manufacturers of this study.

## Data availability statement

The original contributions presented in the study are included in the article/**Supplementary Material**. Further inquiries can be directed to the corresponding author.

## Ethics statement

The studies involving human participants were reviewed and approved by the local ethics committee (Ärztekammer Sachsen-Anhalt, No. 100/21) and is registered by DRKS-ID DRKS00028039. The patients/participants provided their written informed consent to participate in this study.

## Author contributions

JB did study recruitment, performed part of measurements on method comparisons and commutability studies and conceptualized and supervised the direct verification of the reported data. AS performed major parts of the measurements on method comparisons and commutability studies and formal analysis as well as visualization of the data. LD carried out measurements on method comparisons and commutability studies, TM provided resources on analytical equipment and validated the statistical analysis, RD conceptualized medical and ethical aspects of study design and validated the data. CP provided resources and material and validated the reported data. MG conceptualized experiments, carried out formal analysis and data curation and supervised the project. All authors had full access to the data and contributed to the writing and editing of the manuscript. All authors accept responsibility for submission of the manuscript.

## Acknowledgments

The authors would like to thank Sebastian Alers and Marco Reinhart for helpful discussions and critical reading of the manuscript.

## Conflict of interest

Authors JB, AS, LD, RD are employed by MVZ Medizinische Labore Dessau Kassel GmbH. Authors TM and MG are employees of DiaSys Diagnostic GmbH and are named as inventors on a patent application (Deutsche Patentanmeldung 10 2020 122 593.8), claiming the manufacturing and use of the described PETIA for serological quantification of SARS-CoV-2 antigens.

The remaining author declares that the research was conducted in the absence of any commercial or financial relationships that could be construed as a potential conflict of interest.

The PETIA SARS-CoV-2 UTAB FS assay used in this study was kindly provided by DiaSys Diagnostic Systems GmbH, Holzheim, Germany.

## Publisher’s note

All claims expressed in this article are solely those of the authors and do not necessarily represent those of their affiliated organizations, or those of the publisher, the editors and the reviewers. Any product that may be evaluated in this article, or claim that may be made by its manufacturer, is not guaranteed or endorsed by the publisher.
