# Supplementary material for: SARS-CoV-2 antibody progression and neutralizing potential in mild symptomatic COVID-19 patients – a comparative long term post-infection study
Source: Front Immunol. 2022 Aug 17;13:915338. doi: 10.3389/fimmu.2022.915338 (PMC9428854; doi:10.3389/fimmu.2022.915338)

Supplementary material, Brehm *et al.*

Table of Contents

Supplement Table 1: Record of reported Long-COVID symptoms from mild-COVID-19 participants

Supplement Table 2: Information displayed as claimed by manufacturers within respective assay manuals and package inserts

Supplement Table 3: Initial, maximum, minimum and mean antibody levels, detected in COVID-19 patients and vaccinated participants

Supplement Table 4: Comparison of slopes and intercepts for the two groups "COVID-19 participants" vs. "Vaccine" participants

Supplement Figure 1 A: Linear correlation for each immuno-assay, compared to neutralizing antibody titer measured with the TECO-ELISA

Supplement Figure 1 B: Passing & Bablok correlation for each immuno-assay, compared to neutralizing antibody titer measured with the TECO-ELISA

Supplement Figure 2: Area with linear trend TECO inhibition curve of neutralizing antibodies

Supplement Figure 3: Linear correlation among all used immunoassays

Supplement Figure 4: Decline rates in antibody levels, detected in COVID-19 patients and vaccinated participants

Supplement Figure 5: Comparison of the median of the minimum and maximum antibody titer of COVID-19 patients and vaccinated participants

Supplement Table 1: Record of reported Long-COVID symptoms from mild-COVID-19 participants. One participant additionally reported vertigo (No 4). Occurrence of symptoms is indicated by (+) and highlighted in red, no symptoms are indicated by (-). Nearly 70% of participant suffer on symptoms of Long-COVID. Participants also show a broad variety of symptoms and also 55% show persisting symptoms.

		**Long-COVID symptoms**													
												**breathing difficulty**			
**participant No.**	**onset of Long-COVID symptoms after recovery**	**Fever > 38°C**	**persistent cough**	**upper body / chest pain**	**joint pain**	**headache**	**persistent loss of smell and /or taste**	**diarrhea**	**depression**	**lack of concentration**	**fatigue**	**moderate tasks**	**sitting/lying**	**stand up**	**symptoms still present**
1	Ø	-	-	-	-	-	-	-	-	-	-	-	-	-	-
2	Ø	-	-	-	-	-	-	-	-	-	-	-	-	-	-
3	Ø	-	-	-	-	-	-	-	-	-	-	-	-	-	-
4	5-6 months	-	-	-	+	+	-	-	-	-	-	-	-	-	+
5	immediately	-	-	-	-	-	-	+	-	+	+	-	-	-	+
8	immediately	-	-	-	-	+	-	-	-	+	+	-	-	-	+
9	immediately	-	-	+	+	+	-	-	-	+	+	+	-	+	+
12	immediately	-	-	-	-	-	-	-	-	+	+	-	-	-	-
13	immediately	-	-	-	-	-	-	-	-	+	+	-	-	-	+

Supplement Table 2: Information displayed as claimed by manufacturers within respective assay manuals and package inserts.

**Manufacturer**	**DiaSys**	**DiaSorin**	**EUROIMMUN**	**ROCHE**	**ROCHE**	**TECO**
**Assay name**	SARS-CoV-2 UTAB FS	LIAISON® SARS-CoV-2 TrimercS IgG	Anti-SARS-CoV-2 ELISA (IgG)	Elecsys® Anti-SARS-CoV-2	Elecsys® Anti-SARS-CoV-2 S	SARS-CoV-2 Neutralizing Antibody Assay
**Method**	PETIA	CLIA	ELISA	ECLIA	ECLIA	ELISA
**Immunoglobulin**	IgG / IgM / IgA	IgG	IgG	IgG / IgM / IgA	IgG / IgM / IgA	IgG / IgM / IgA
**Antigen**	Spike receptor binding domain (RBD)	S1 and S2 domain of spike protein (S1/S2)	S1 domain of spike protein, including RBD	Nucleocapsid protein (N)	Spike receptor binding domain (RBD)	Spike receptor binding domain (RBD)
**Version/Lot**	E113/21 E114/21	135479	E210603CB	55891800	54861700	210501-HW
**Quantification**	quantitative	quantitative	quantitative	qualitative	quantitative	quantitative
**Calibrator**	5-level, liquid (Lot: E106-E110)	2-level, liquid (Lot: 135479)	6-level, liquid (Lot: E210603CB)	2-level, liquid (Lot: 558918)	2-level, liquid (Lot: 513360)	5-level, liquid
**Controls**	2-level, liquid (Lot: 31255/56)	2-level, liquid (Lot: 135590)	2-level, liquid (Lot: E210603CB)	2-level, liquid (Lot: 543132/34)	2-level, liquid (Lot: 543174/75)	2-level, liquid (Lot: 210501-HW)
**sample (µl)**	13	20	10	20	20	10
**TtR (min)**	11	35	150	18	18	120

Supplement Figure 1 A: Linear correlation for each immuno-assay, compared to neutralizing antibody titer measured with the TECO-ELISA. Results for Roche N-Test are only semi-quantitative as cut-off-index (COI), N =83.

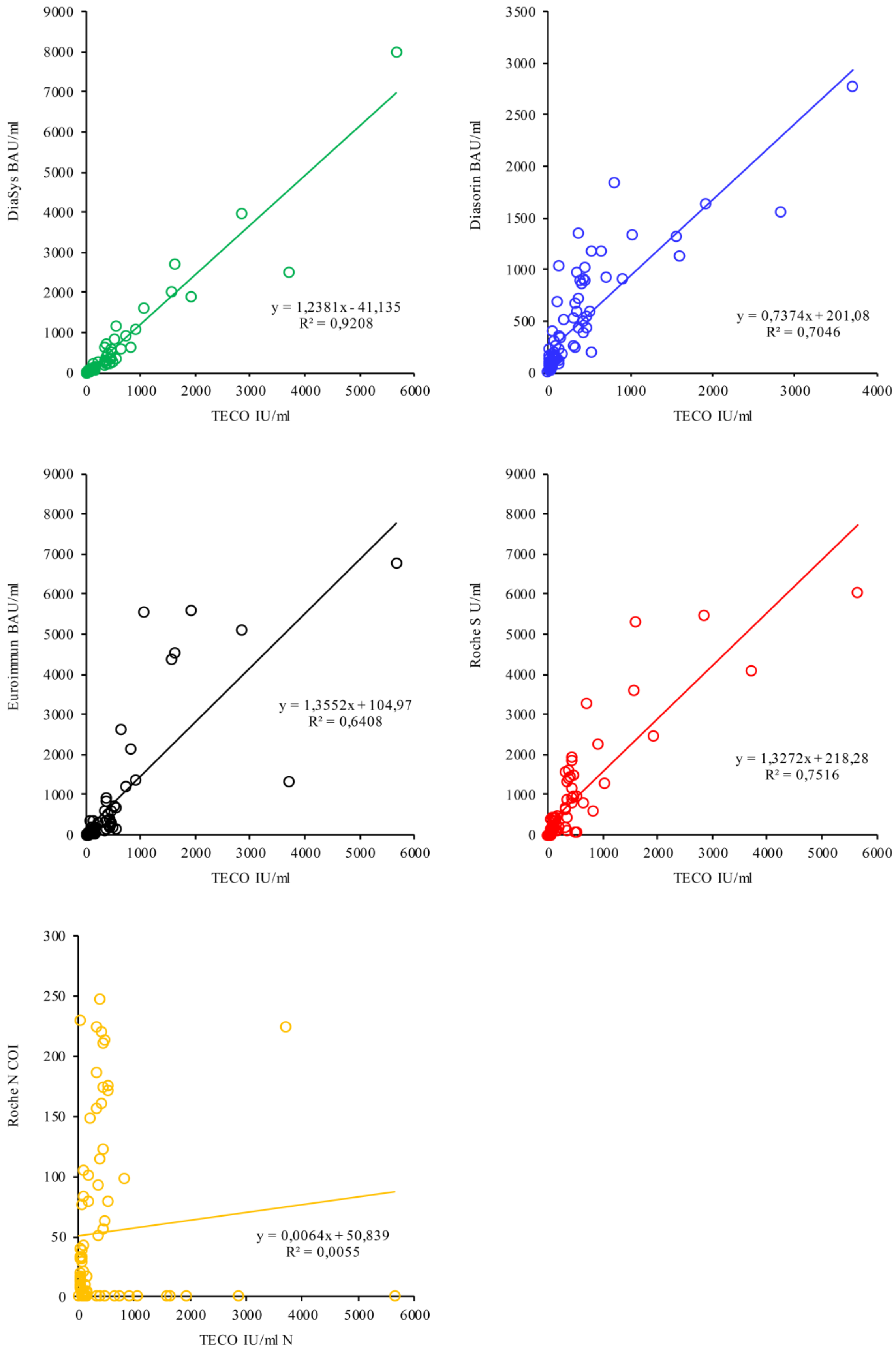

Supplement Figure 1 B: Passing & Bablok correlation for each immuno-assay, compared to neutralizing antibody titer measured with the TECO-ELISA. Additional evaluation of corresponding immuno-assays, compared to the neutralizing antibody TECO-ELISA by Passing & Bablok indicating Spearman coefficient R. The best correlation is shown by DiaSys against TECO (R = 0.967), followed by EUROIMMUN (R = 0.921), Roche S (R = 0.908), DiaSorin (R = 0.879) and Roche N (R = 0.145).

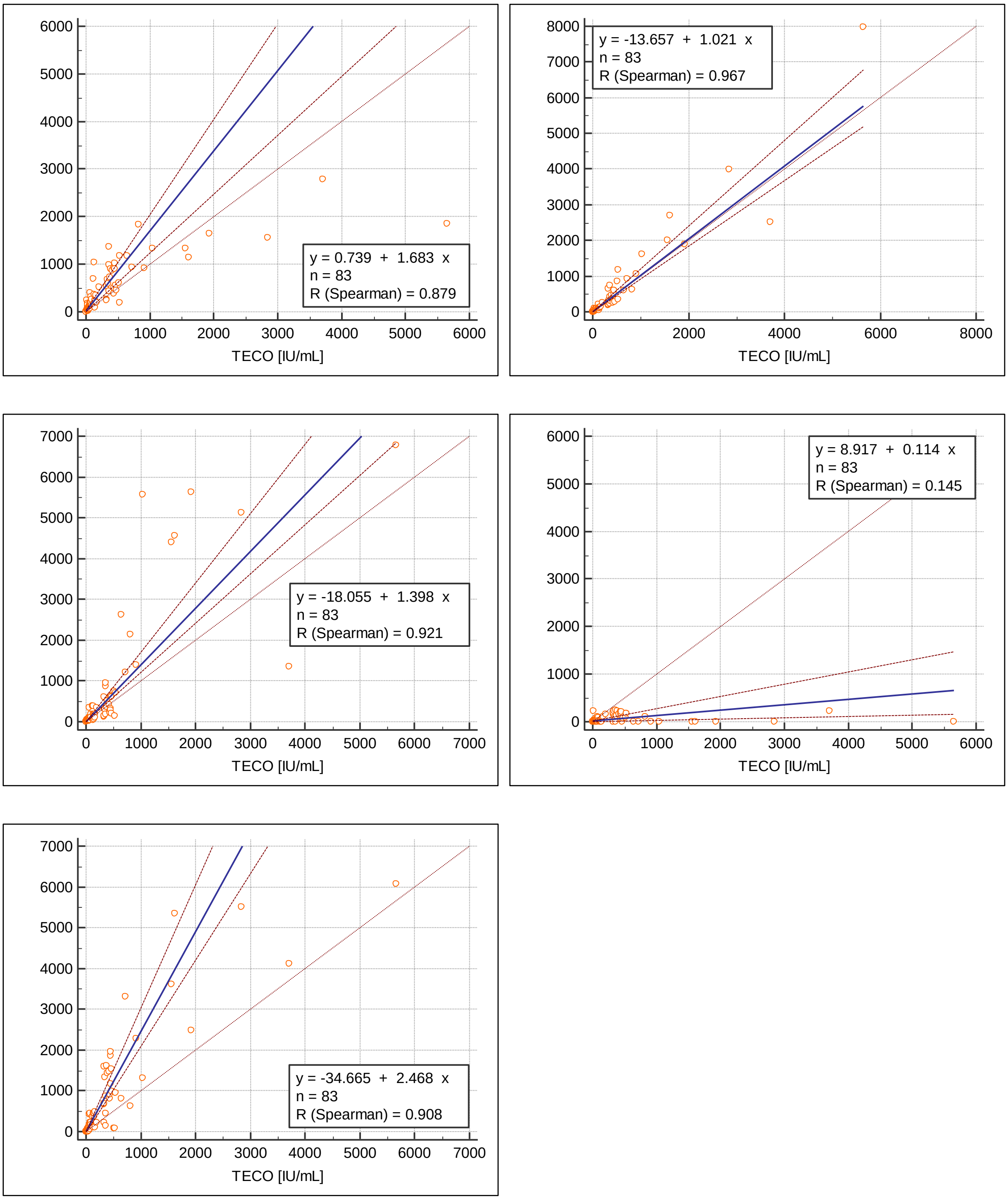

Supplement Figure 2: Area with linear trend (green line) in the TECO inhibition curve of neutralizing antibodies, in dependence of respective measured antibody concentration. Cut-off is indicated as red line.

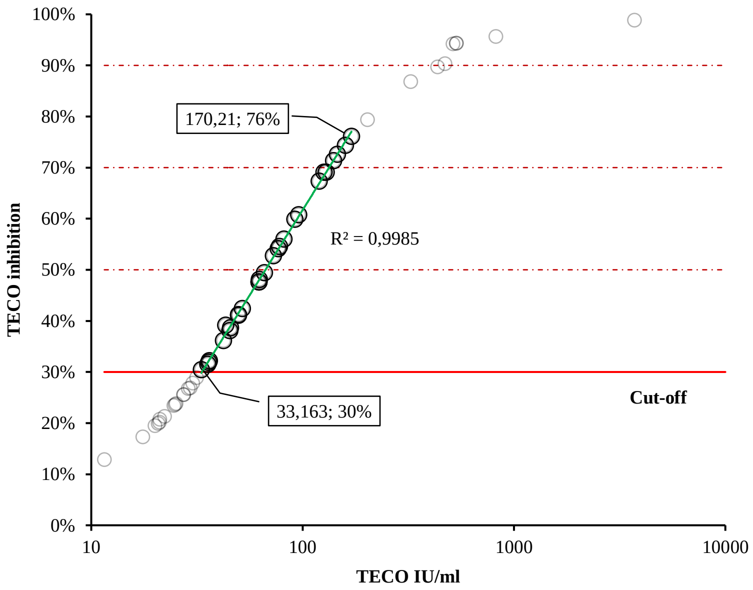

Supplement Figure 3: Linear correlation among all used immunoassays (except TECO neutralization test), to assess intra-assay agreement of results. Note that a linear correlation, due to the distribution of samples, accumulating at the lower range (0-1000 BAU/ml) need to be considered with care. Correlations are represented by two-colored circles, in regard to the original color-code of the individual sero-assays (Supplement Figure 1).

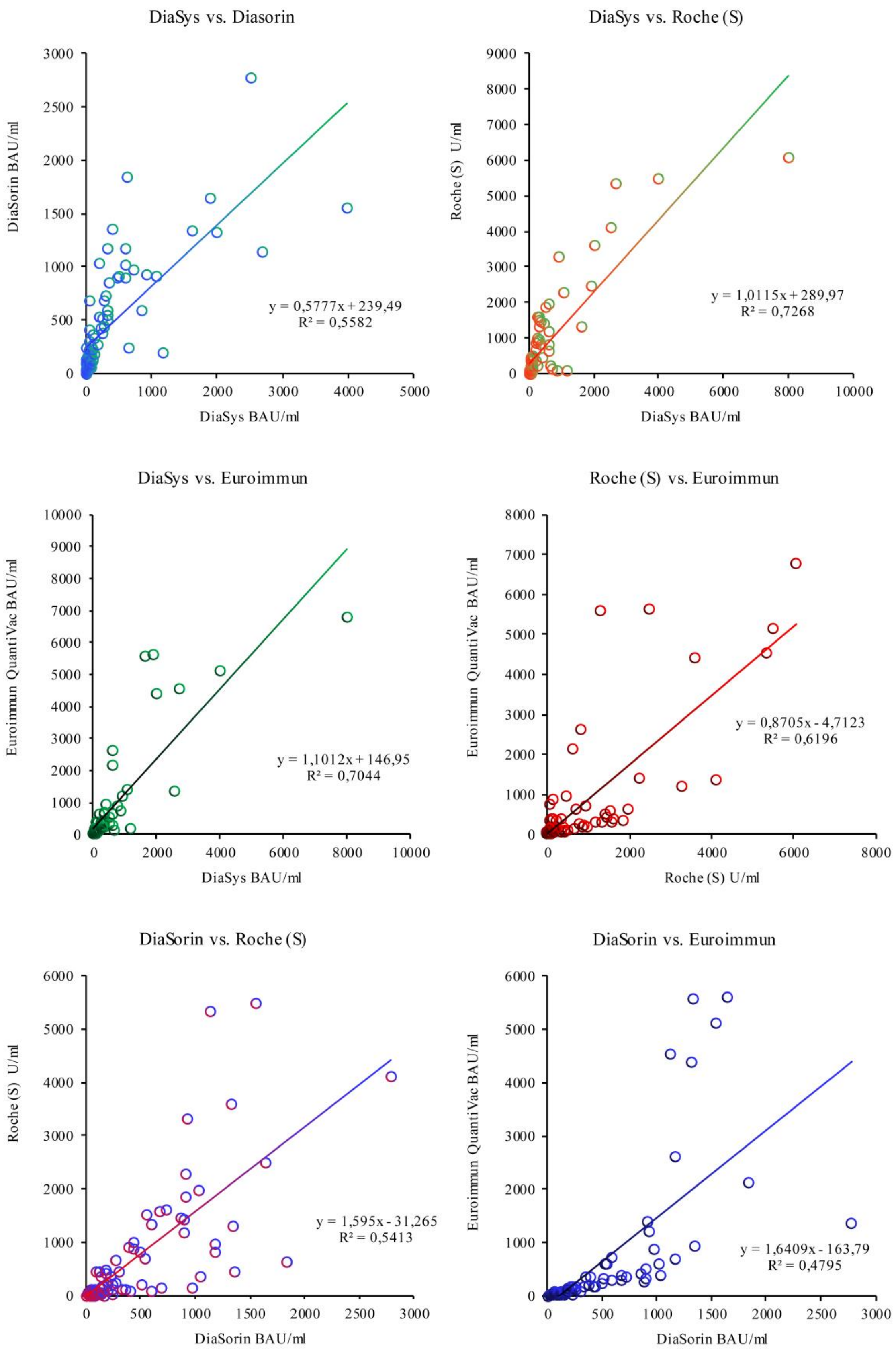

Supplement Table 3: Initial, maximum, minimum and mean antibody levels, detected in COVID-19 patients and vaccinated participants. Grey shaded participant represent individual humoral antibody immune response of participants (No. 1 to 13), recovered from COVID-19 and monitored over respective days with different immuno-assays. Participant No. 14 to 20 represent individual humoral antibody immune response of vaccinated participants, monitored over respective days post vaccination with different immuno-assays. "Max. antibody level" indicates the maximal increase from the "Initial antibody value"/first measurement. "Min. antibody level" indicates the maximal decrease in respect to the "Max. antibody level". For mater of clarity, only data obtained from Euroimmun (BAU/ml) and TECO neutralization assay (IU/ml) are listed for direct comparison. Note that, despite the drop in antibody levels, it should be noted and discussed in the text that the minimum antibody levels in the vaccinated participants seem to be much higher than the minimum antibody levels in the infected participants. Please perform statistical analysis for the differences seen in the minimum antibody levels among the two groups.

Participant No.		Unit	Initial antibody level	Max. antibody level	Min. antibody level	Mean antibody level (IU/ml)
1		BAU/ml	40.9	178.2 (435 %)	49.4 (28 %)	81.9 ± 51.5
		IU/ml	42.2	145.6 (345 %)	119.4 (82 %)	112.0 ± 38.4
2		BAU/ml	16.0	19.8 (123 %)	15.3 (77 %)	17.8 ± 2.0
		IU/ml	19.9	24.8 (124 %)	20.6 (83 %)	21.7 ± 1.9
3		BAU/ml	168.2	168.2 (0 %)	20.4 (12%)	66.5 ± 69.6
		IU/ml	159.3	159.3 (0 %)	25.1 (16 %)	64.5 ± 57.2
4		BAU/ml	150.4	243.2 (161 %)	91.8 (38 %)	153.2 ± 52.7
		IU/ml	531.74	531.7 (0 %)	170.2 (32 %)	358.6 ± 121.1
5		BAU/ml	45.1	79.6 (176 %)	27.8 (34 %)	45.5 ± 24.1
		IU/ml	45.2	62.0 (137 %)	43.1 (69 %)	50.6 ± 8.5
6		BAU/ml	51.5	51.5 (0 %)	15.3 (29 %)	33.4 ± 17.9
		IU/ml	24.5	31.4 (128 %)	28.6 (91 %)	28.7 ± 3.0
7		BAU/ml	30.4	30.4 (0 %)	17.6 (57 %)	27.8 ± 8.3
		IU/ml	35.5	49.6 (139 %)	29.3 (59 %)	41.0 ± 10.3
8		BAU/ml	873.6	873.6 (0 %)	104.3 (12 %)	319.1 ± 371.7
		IU/ml	361.2	361.2 (0 %)	65.8 (18%)	147.8 ± 142.7
9		BAU/ml	739.2	739.2 (0 %)	326.4 (44 %)	548.0 ± 175.2
		IU/ml	514.4	514.4 (0 %)	391.3 (76 %)	450.9 ± 50.5
10		BAU/ml	22.4	39.0 (174 %)	14.1 (36 %)	22.2 ± 10.1
		IU/ml	62.0	62.0 (0 %)	21.0 (34 %)	36.5 ± 15.6
11		BAU/ml	2147.2	2147.2 (0 %)	316.8 (15 %)	790.4 ± 773.0
		IU/ml	818.4	818.4 (0 %)	332.1 (40 %)	494.2 ± 196.4

12		BAU/ml	65.6	65.6<br>(0 %)	47.6<br>(72 %)	54.4 ± 9.8
		IU/ml	45.5	81.5<br>(179 %)	62.4<br>(76 %)	63.2 ± 18.0
13		BAU/ml	336.9	336.9<br>(0 %)	194.8<br>(58 %)	265.5 ± 68.8
		IU/ml	202.5	471.3<br>(232 %)	434.4<br>(92 %)	363.7 ± 119.5
14		BAU/ml	6.72	947.2<br>(> 1000 %)	104<br>(11 %)	294.8 ± 394.3
		IU/ml	0.01	365.1<br>(> 1000 %)	76.4<br>(20 %)	115.8 ± 148.4
15		BAU/ml	3.2	6771.2<br>(> 1000 %)	601.6<br>(8 %)	3248.8 ± 3443.2
		IU/ml	0.94	5656.4<br>(> 1000 %)	464.0<br>(8 %)	2011.7 ± 2564.5
16		BAU/ml	3.84	5574.4<br>(> 1000 %)	179.2<br>(3 %)	2095.4 ± 2609.6
		IU/ml	0.01	1037.6<br>(> 1000 %)	143.25<br>(13 %)	457.1 ± 476.4
17		BAU/ml	3.84	4390.4<br>(> 1000 %)	608<br>(13 %)	1346.6 ± 2044.5
		IU/ml	0.24	1560.5<br>(> 1000 %)	324.6<br>(20 %)	501.2 ± 718.9
18		BAU/ml	4.48	5126.4<br>(> 1000 %)	1401.6<br>(27 %)	1727.4 ± 2341.8
		IU/ml	0.49	2844.3<br>(> 1000 %)	909.5<br>(32 %)	971.4 ± 1311.5
19		BAU/ml	5.12	4550.4<br>(> 1000 %)	1203.2<br>(26 %)	1526.9 ± 2077.7
		IU/ml	0.01	1615.4<br>(> 1000 %)	719.2<br>(44 %)	599.7 ± 751.1
20		BAU/ml	18.56	1360.0<br>(> 1000 %)	278.4<br>(20 %)	552.3 ± 711.4
		IU/ml	17.5	3706.4<br>(> 1000 %)	446.5<br>(12 %)	1390.1 ± 2017.4

Supplement Figure 4: Decline rates in antibody levels, detected in COVID-19 patients and vaccinated participants. Antibody levels of TECO NT method (IU/mL) were calculated in %, setting the highest initial value to 100%. Concentration values of the measuring times before highest concentration were not considered. The two regression lines clearly differ (slopes P = 0.009503, intercepts P = 0.006324, see also Supplement Table 4), indicating, that the concentrations of the vaccinated patients rapidly decrease over time. On the contrary, concentrations of the COVID-19 patients are lower at the beginning but they remain constant in general during the time. COVID-19 patients are indicated by a blue line and empty circles, vaccinated participants are indicated by green line and red squares.

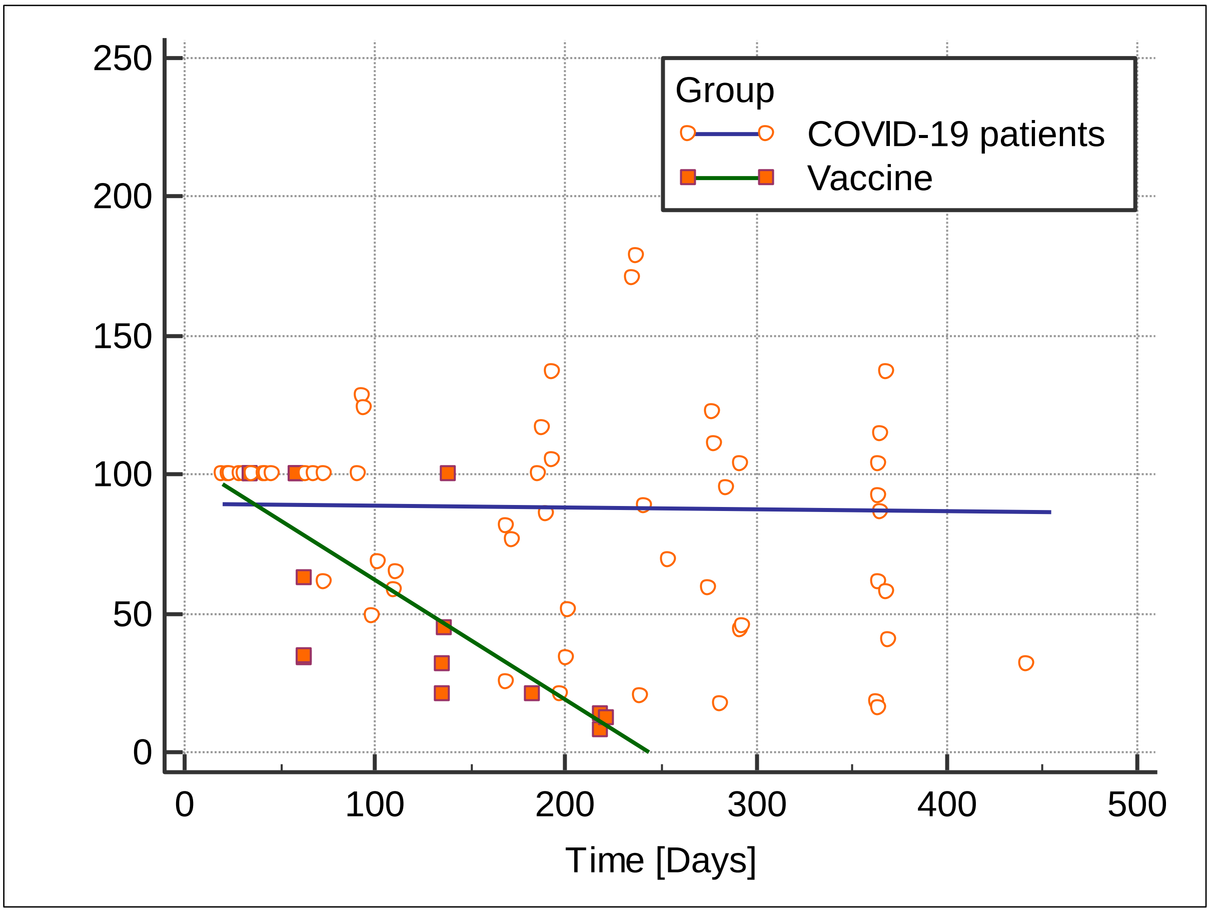

Supplement Table 4: Comparison of slopes and intercepts for the two groups ”COVID-19 participants” vs. “Vaccine” participants. The regression equations are reported as first for the two groups. The intercept and slope unit are given as percentage, as the sample concentrations were transformed by setting the highest initial value to 100% (see also Supplement Table 3 and Supplement Figure 4). The comparison of slopes, shown in the second part of the table, displays a statistically significant difference (P = 0.009503), as the concentrations of the subgroup “Vaccine” decrease over the time on the contrary of the concentrations of the “COVID-19 participants” subgroup. The latter shows lower initial concentrations, indicated by the different intercepts (intercepts P = 0.006324, third part of the table). Antibody values reported in % need to be interpreted with care, as absolute differences among values are not addressed. For a direct comparison of the two groups, reported in IU/ml please refer to Supplement Figure 5A and B.

**Subgroup: COVID-19 participants**	
y = 89.4962 -0.007127 x	
**Subgroup: Vaccine**	
y = 105.1128 -0.4317 x	
**Comparison of slopes**	
Difference	0.4246
Standard error	0.1592
t	2.6669
DF	70
P	0.009503
**Common slope**	
Slope	-0.04065
Standard error	0.04293
**Comparison of intercepts**	
Adjusted difference	34.1204
Standard error	12.1196
t	2.8153
DF	70
P	0.006324

Supplement Figure 5A and 5B: Comparison of the median of the minimum (A) and maximum (B) antibody titer of COVID-19 patients and vaccinated participants. The values for the surrogate neutralizing test from the TECO assay have been used for the assessment. Despite the drop in percentage of antibody levels in vaccinated participants (Supplement Table 3 and Supplement Figure 4), the absolute minimum and maximum antibody levels in this group are much higher, compared to the corresponding levels in infected participants. The median of each group is reported by a box-whisker plot. The p-value of the Mann-Whitney test (independent samples) for the respective medians is statistically significant between COVID-19 patients and vaccinated participants for both the concentrations, minimum (p = 0.0079) and maximum (p = 0.001).

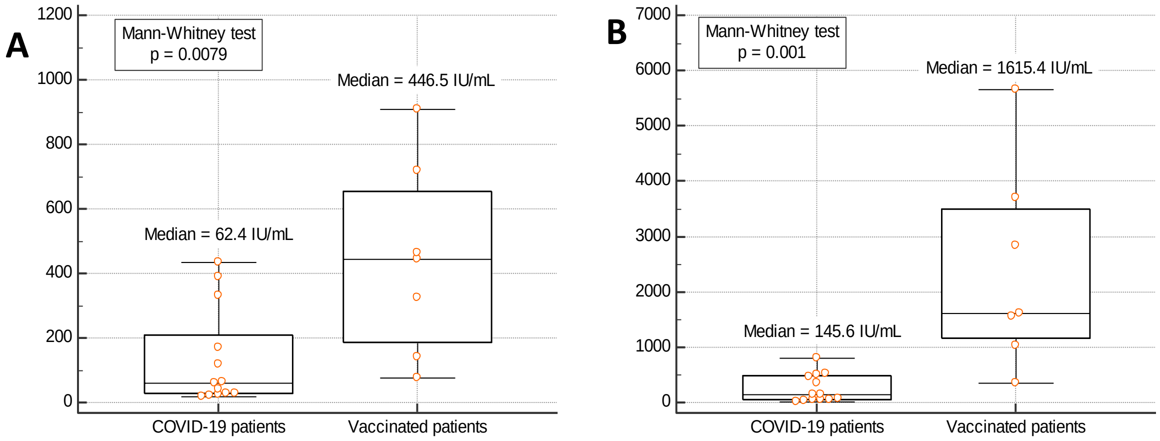

Supplement: Supplementary file 1 [file DataSheet_1.pdf]
